# Supplementary material for: TDP-43 forms amyloid filaments with a distinct fold in type A FTLD-TDP
Source: Nature. 2023 Aug 2;620(7975):898–903. doi: 10.1038/s41586-023-06405-w (PMC10447236; doi:10.1038/s41586-023-06405-w)
Supplement: Supplementary file 1 — Supplementary Information [file 41586_2023_6405_MOESM1_ESM.pdf]

nature portfolio

https://doi.org/10.1038/s41586-023-06405-w

Supplementary information

TDP-43 forms amyloid filaments with a distinct fold in type A FTLD-TDP

In the format provided by the
authors and unedited

Supplementary Figure 1| Gel source data

ED Fig 1c

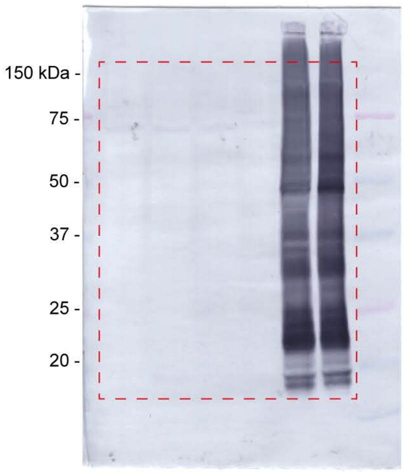

Anti-phosphorylated
S409 and S410 TDP-43

ED Fig 1c

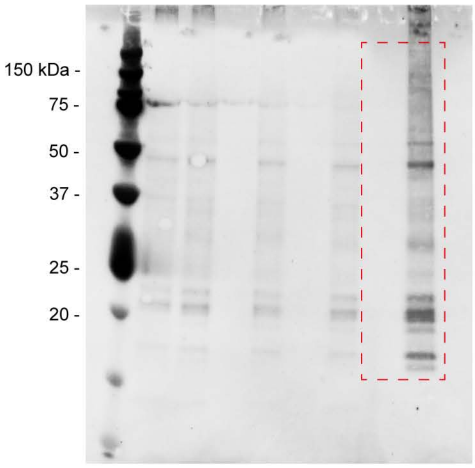

Anti-phosphorylated
S409 and S410 TDP-43

Supplementary Data Table 1| TMEM106B peptide sequences identified by mass spectrometry

	Peptide count		
Peptide	Individual 1	Individual 2	Individual 3
^{107}LLSGLAVF^{114}	-	1	-
^{126}IGVKS^{130}	-	2	-
^{126}IGVKSAYVSY^{135}	2	-	5
^{133}VSYDVQKRTIY^{143}	20	-	-
^{136}DVQKRTIY^{143}	-	9	-
^{136}DVQKRTIYL^{144}	-	1	-
^{159}SVEVENITAQVQF^{171}	-	-	-
^{191}DMKQIDY^{197}	-	26	-
^{191}DMKQIDYTVPTVIAEEMSY^{209}	-	1	-
^{198}TVPTVIAEEMSY^{209}	16	19	-
^{198}TVPTVIAEEMSYMY^{211}	1	-	-
^{210}MYDFCTL^{216}	-	4	-
^{227}MMQVTVTTTY^{236}	-	11	-
^{237}FGHSEQISQERY^{248}	29	22	59
^{238}GHSEQISQERY^{248}	3	6	-
^{238}GHSEQISQERYQY^{250}	1	-	-

Supplementary Data Table 2| Compatibility of *TARDBP* mutations with the chevron and double-spiral TDP-43 filament folds

Mutation	Compatible with the chevron fold?	Compatible with the double-spiral fold?
G287S	Yes	Yes
G290A	Yes	Yes
S292N	Yes	Yes
G294A	No	Yes
G294V	No	Yes
G295S	Yes*	Yes
G295R	Yes*	Yes
G298S	No	No
M311V	Yes	Yes
A315T	No	Yes
A315E	No	Yes
A321G	Yes	Yes
Q331K	No	No
S332N	No	No
G335D	No	No
M337V	Yes	Yes
Q343R	Yes**	No
N345K	Yes	No
G348C	No	Yes
G348V	No	Yes
G348R	No	Yes
N352S	Yes	Yes
G357R	Yes	Yes
G357S	Yes	Yes

*Compatible only with the alternative conformation of the N-terminal region

**Compatible only with the main conformation of the L4-L5 turn
