# Supplementary material for: TDP-43 forms amyloid filaments with a distinct fold in type A FTLD-TDP
Source: Nature. 2023 Aug 2;620(7975):898–903. doi: 10.1038/s41586-023-06405-w (PMC10447236; doi:10.1038/s41586-023-06405-w)
Supplement: Supplementary file 2 — Reporting Summary [file 41586_2023_6405_MOESM2_ESM.pdf]

nature portfolio

Corresponding author(s): Benjamin Ryskeldi-Falcon

Last updated by author(s): Jul 17, 2023

Reporting Summary

Nature Portfolio wishes to improve the reproducibility of the work that we publish. This form provides structure for consistency and transparency in reporting. For further information on Nature Portfolio policies, see our Editorial Policies and the Editorial Policy Checklist.

Statistics

For all statistical analyses, confirm that the following items are present in the figure legend, table legend, main text, or Methods section.

n/a	Confirmed	
[ ]	[x]	The exact sample size (*n*) for each experimental group/condition, given as a discrete number and unit of measurement
[ ]	[x]	A statement on whether measurements were taken from distinct samples or whether the same sample was measured repeatedly
[x]	[ ]	The statistical test(s) used AND whether they are one- or two-sided<br>*Only common tests should be described solely by name; describe more complex techniques in the Methods section.*
[x]	[ ]	A description of all covariates tested
[x]	[ ]	A description of any assumptions or corrections, such as tests of normality and adjustment for multiple comparisons
[ ]	[x]	A full description of the statistical parameters including central tendency (e.g. means) or other basic estimates (e.g. regression coefficient) AND variation (e.g. standard deviation) or associated estimates of uncertainty (e.g. confidence intervals)
[x]	[ ]	For null hypothesis testing, the test statistic (e.g. *F*, *t*, *r*) with confidence intervals, effect sizes, degrees of freedom and *P* value noted<br>*Give P values as exact values whenever suitable.*
[x]	[ ]	For Bayesian analysis, information on the choice of priors and Markov chain Monte Carlo settings
[x]	[ ]	For hierarchical and complex designs, identification of the appropriate level for tests and full reporting of outcomes
[x]	[ ]	Estimates of effect sizes (e.g. Cohen's *d*, Pearson's *r*), indicating how they were calculated

Our web collection on statistics for biologists contains articles on many of the points above.

Software and code

Policy information about availability of computer code

Data collection	EPU 2.14
Data analysis	Mascot 2.4, Scaffold 4.0, RELION 4.0, CTFFIND 4.1, COOT 0.9.8.2, ISOLDE 1.5, REFMAC 5.8.0387, Servalcat 0.3.0, ChimeraX 1.5, MolProbity 4.5.2.

For manuscripts utilizing custom algorithms or software that are central to the research but not yet described in published literature, software must be made available to editors and reviewers. We strongly encourage code deposition in a community repository (e.g. GitHub). See the Nature Portfolio guidelines for submitting code & software for further information.

Data

Policy information about availability of data

All manuscripts must include a data availability statement. This statement should provide the following information, where applicable:

- Accession codes, unique identifiers, or web links for publicly available datasets
- A description of any restrictions on data availability
- For clinical datasets or third party data, please ensure that the statement adheres to our policy

Whole-exome data have been deposited in the National Institute on Ageing Alzheimer’s Disease Data Storage Site (NIAGADS), under accession code NG00107. Mass spectrometry data have been deposited to the Proteomics Identifications (PRIDE) database under the accession numbers PXD040102 and PDX043731. Cryo-EM datasets have been deposited to the Electron Microscopy Public Image Archive (EMPIAR) under accession codes EMPIAR-11438 for individual 1, EMPIAR-11428 for

individual 2 and EMPIAR-11429 for individual 3. Cryo-EM maps have been deposited to the Electron Microscopy Data Bank (EMDB) under accession codes EMD-16628 for individual 1, variant 1; EMD-16642 for individual 1, variant 2; EMD-16643 for individual 1, variant 3; EMD-16677 for individual 2, variant 1; EMD-16681 for individual 2, variant 2; and EMD-16682 for individual 3, variant 1. Atomic models have been deposited to the Protein Data Bank (PDB) under accession codes 8CG3 for variant 1, 8CGG for variant 2 and 8CGH for variant 3. The atomic model of TDP-43 filaments from ALS and type B FTLD-TDP is available at the PDB under accession number 7PY2.

Human research participants

Policy information about studies involving human research participants and Sex and Gender in Research.

Reporting on sex and gender	3 females and 2 males.
Population characteristics	See Extended Data Table 1. Between 50 and 75 years-of-age. Disease associated mutations in GRN. Wild-type C9orf. Clinical diagnoses of FTD. Neuropathological diagnoses of type A FTLD-TDP.
Recruitment	Selected based on availability and neuropathological examination.
Ethics oversight	Human tissue samples were from the Brain Library of the Dementia Laboratory at Indiana University School of Medicine (individuals 2, 4, and 5) and the Manchester Brain Bank (individuals 1 and 3). Their use in this study was approved by the ethical review processes at each institution. Informed consent was obtained from the patients' next of kin.

Note that full information on the approval of the study protocol must also be provided in the manuscript.

Field-specific reporting

Please select the one below that is the best fit for your research. If you are not sure, read the appropriate sections before making your selection.

☒ Life sciences ☐ Behavioural & social sciences ☐ Ecological, evolutionary & environmental sciences

For a reference copy of the document with all sections, see nature.com/documents/nr-reporting-summary-flat.pdf

Life sciences study design

All studies must disclose on these points even when the disclosure is negative.

Sample size	Prefrontal cortex from 3 individuals with type A FTLD-TDP. Samples were chosen based on availability and neuropathological examination.
Data exclusions	Pre-established common image classification procedures (Scheres 2012. J. Struc. Biol. 180, 519-530) were employed to select particle images with the highest resolution content in the cryo-EM reconstruction process. Details of the number of selected images are given in Extended Data Table 2.
Replication	All attempts at replication were successful. At least three independent biological repeats per experiment where representative data are shown, as described in the main text.
Randomization	Randomisation was not performed. As the samples were limited by brain availability, randomisation would not have reduced any bias in this study.
Blinding	The investigators were not blinded to allocation during experiments and outcome assessment. The perceived risk of detection/performance bias was deemed negligible.

Reporting for specific materials, systems and methods

We require information from authors about some types of materials, experimental systems and methods used in many studies. Here, indicate whether each material, system or method listed is relevant to your study. If you are not sure if a list item applies to your research, read the appropriate section before selecting a response.

Materials & experimental systems

n/a	Involved in the study
☐	☒ Antibodies
☒	☐ Eukaryotic cell lines
☒	☐ Palaeontology and archaeology
☒	☐ Animals and other organisms
☒	☐ Clinical data
☒	☐ Dual use research of concern

Methods

n/a	Involved in the study
☒	☐ ChIP-seq
☒	☐ Flow cytometry
☒	☐ MRI-based neuroimaging

Antibodies

Antibodies used

The primary antibodies used were anti-phospho S409 and S410 TDP-43 (Cosmo Bio USA, CAC-TIP-PTD-M01, clone 11-9), anti-N-terminus TDP-43 (Abcam, ab57105) and anti-N-terminus TDP-43 (Proteintech, 10782-2-AP).

Validation

Validation of anti-phospho S409 and S410 TDP-43 against human phospho S409 and S410 TDP-43 for histology and immunoblotting is presented in the manufacturer's datasheet (Cosmo Bio USA) and in (Inukai et al. 2008. FEBS Lett. 582, 2899-2904). Validation of anti-N-terminus TDP-43 against human TDP-43 for histology and immunoblotting is presented in the manufacturers' datasheets (Abcam and Proteintech).
